# A prehospital diagnostic algorithm for strokes using machine learning: a prospective observational study

**DOI:** 10.1038/s41598-021-99828-2

**Published:** 2021-10-15

**Authors:** Yosuke Hayashi, Tadanaga Shimada, Noriyuki Hattori, Takashi Shimazui, Yoichi Yoshida, Rie E. Miura, Yasuo Yamao, Ryuzo Abe, Eiichi Kobayashi, Yasuo Iwadate, Taka-aki Nakada

**Affiliations:** 1 Department of Emergency and Critical Care Medicine, Chiba University Graduate School of Medicine, 1-8-1 Inohana, Chiba, 260-8677 Japan; 2 Department of Neurological Surgery, Chiba University Graduate School of Medicine, Chiba, Japan; 3 Smart119 Inc, Chiba, Japan

**Keywords:** Stroke, Outcomes research

## Abstract

High precision is optimal in prehospital diagnostic algorithms for strokes and large vessel occlusions. We hypothesized that prehospital diagnostic algorithms for strokes and their subcategories using machine learning could have high predictive value. Consecutive adult patients with suspected stroke as per emergency medical service personnel were enrolled in a prospective multicenter observational study in 12 hospitals in Japan. Five diagnostic algorithms using machine learning, including logistic regression, random forest, support vector machine, and eXtreme Gradient Boosting, were evaluated for stroke and subcategories including acute ischemic stroke with/without large vessel occlusions, intracranial hemorrhage, and subarachnoid hemorrhage. Of the 1446 patients in the analysis, 1156 (80%) were randomly included in the training (derivation) cohort and cohorts, and 290 (20%) were included in the test (validation) cohort. In the diagnostic algorithms for strokes using eXtreme Gradient Boosting had the highest diagnostic value (test data, area under the receiver operating curve 0.980). In the diagnostic algorithms for the subcategories using eXtreme Gradient Boosting had a high predictive value (test data, area under the receiver operating curve, acute ischemic stroke with/without large vessel occlusions 0.898/0.882, intracranial hemorrhage 0.866, subarachnoid hemorrhage 0.926). Prehospital diagnostic algorithms using machine learning had high predictive value for strokes and their subcategories.

## Introduction

Stroke is an acute life-threatening disease that mostly occurs in out-of-hospital settings^1^. Emergency medical services (EMS) personnel, who are the first healthcare providers to respond to patients with a suspected stroke, evaluate the risk of stroke and transport those patients to hospitals. Early initiation of therapeutic approaches, including endovascular therapy for large vessel occlusion (LVO)^2,3^ is key to improving the clinical outcomes of strokes^4^. Thus, higher precision of stroke prediction in prehospital settings may contribute to improving the quality of stroke care and clinical outcomes.

Prehospital diagnostic algorithms for strokes, which are also known as stroke scales, have been developed substantially^5–7^. There could be a propensity to place importance on limiting the number of predictive values for simplification rather than enhancing predictive ability in the prediction precision for strokes or LVOs (area under the receiver operating characteristic curve [AUROC], stroke prediction, Cincinnati Prehospital Stroke Scale [CPSS] 0.813^5^, Japan Urgent Stroke Triage [JUST] Score 0.800–0.88^6^; LVO prediction, 8 prehospital stroke scales 0.72–0.83^7^). Recent advances in statistical approaches using machine learning have significantly improved the precision of predicting algorithms for acute diseases by using multiple predictive factors, including out-of-hospital cardiac arrest, acute coronary syndrome, and sepsis^8–10^. However, few investigations have focused on prehospital stroke/LVO diagnostic algorithms using machine learning.

Therefore, we tested the hypothesis that prehospital stroke diagnostic algorithms using machine learning had a high predictive value. We prospectively enrolled a large cohort of patients with suspected stroke in prehospital settings and analyzed them using five machine learning algorithms.

## Results

### Baseline characteristics and outcomes

In a training cohort as a derivation cohort, 834 patients had a stroke (training cohort, Table 1). Patients with strokes had significantly increased age, increased past history of heart diseases (atrial fibrillation and hypertension), decreased past history of diabetes mellitus, and decreased past history of neurological diseases compared to non-stroke patients. In terms of vital signs, patients with strokes had decreased heart rates, increased arrhythmia, increased blood pressure, and decreased probability of impaired Glasgow coma scale (GCS) components compared to non-stroke patients. Patients with strokes had decreased dizziness and convulsion and increased upper limb paralysis, hemiparalysis, facial palsy, and dysarthria compared to non-stroke patients. Onset timing (hourly) was earlier in patients with strokes than in those without strokes**.** Similar differences were observed in the test cohort data as a validation cohort (test cohort, Supplementary Table S2).

**Table 1:** Baseline characteristics and clinical outcomes in the training cohort.

	Stroke (n = 834)	Non-stroke (n = 322)	*P* value
Age, years	74.0 (65.0–82.0)	72.0 (57.2–81.0)	0.004
Male sex, n (%)	507 (60.8)	182 (56.5)	0.208
**Past medical history**			
Atrial fibrillation, n (%)	70 (8.4)	14 (4.3)	0.003
Hypertension, n (%)	380 (45.6)	138 (42.9)	0.017
Diabetes mellitus, n (%)	109 (13.1)	53 (16.5)	0.015
Intracranial hemorrhage, n (%)	38 (4.6)	27 (8.4)	< 0.001
Cerebral infarction, n (%)	157 (18.8)	62 (19.3)	0.008
Epilepsy, n (%)	6 (0.7)	13 (4.0)	< 0.001
Psychiatric disorder, n (%)	21 (2.5)	23 (7.1)	< 0.001
**Vital signs**			
Heart rate	82 (70–96)	84 (74–98)	0.033
Arrhythmia, n (%)	192 (23.0)	37 (11.5)	< 0.001
Systolic blood pressure	174 (155–200)	160(140–180)	< 0.001
Diastolic blood pressure	97(83–114)	90 (79–104)	< 0.001
Body temperature	36.5 (36.2–36.8)	36.5 (36.2–36.8)	0.725
Japan Coma scale = 0, n (%)	357 (42.8)	170 (52.8)	0.007
Glasgow Coma scale			
Eye opening = 4, n (%)	627 (75.2)	269 (83.5)	0.019
Best verbal response = 5, n (%)	402 (48.2)	188 (58.4)	0.003
Best motor response = 6, n (%)	608 (72.9)	257 (79.8)	0.018
**Symptoms**			
Vomiting, n (%)	135 (16.2)	38 (11.8)	0.114
Dizziness, n (%)	49 (5.9)	46 (14.3)	< 0.001
Convulsion, n (%)	15 (1.8)	40 (12.4)	< 0.001
Upper limbs paralysis, n (%)	354 (42.4)	115 (35.7)	0.043
Lower limbs paralysis, n (%)	431 (51.7)	152 (47.2)	0.194
Hemiparalysis, n (%)	198 (23.7)	32 (9.9)	< 0.001
Conjugate deviation, n (%)	90 (10.8)	21 (6.5)	0.064
Visual field defects, n (%)	14 (1.7)	4 (1.2)	0.228
Facial palsy, n (%)	55 (26.3)	10 (12.3)	0.036
Ataxia, n (%)	23 (11.0)	8 (9.9)	0.824
Sensory impairment, n (%)	29 (13.9)	4 (4.9)	0.066
Aphasia, n (%)	69 (33.0)	22 (27.2)	0.411
Dysarthria, n (%)	69 (33.0)	11 (13.6)	0.001
Unilateral spatial neglect, n (%)	29 (3.5)	6 (1.9)	0.138
Onset timing Monday, n (%)	136 (16.3)	40 (12.4)	0.120
Onset timing (h)	12 (7–18)	14 (8–19)	0.023
Minimum THI	13.1 (7.7–18.7)	12.2 (8.2–18.8)	0.849

*JCS* Japan coma scale, *GCS* Glasgow coma scale, *THI* thermo-hydrological index.

Data are presented as median and interquartile range for continuous variables.

*P*-values were calculated using Pearson’s chi-square test or the Mann–Whitney U test.

### Prediction of stroke

**Figure 1:**
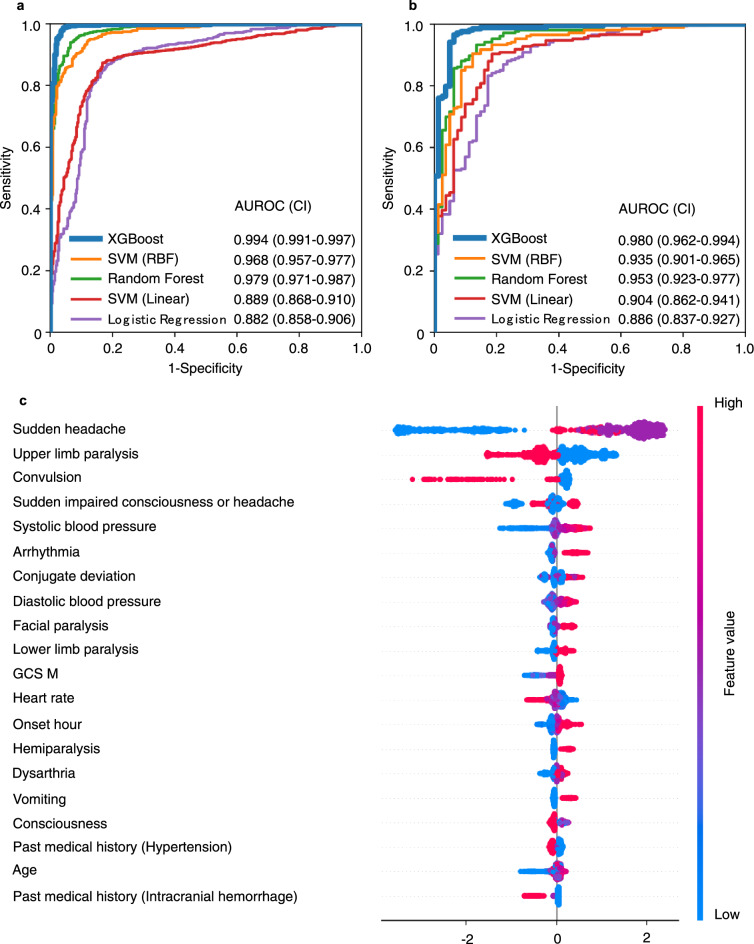
Receiver operating characteristic curve and the SHAP value of prehospital stroke prediction. (**a**) Training cohort (derivation cohort). (**b**) Test cohort (validation cohort). (**c**) SHAP value of stroke. AUROC (area under the receiver operating characteristic curve), CI (confidence interval), XGBoost (eXtreme Gradient Boosting), SVM (support vector machine), SHAP (SHapley Additive exPlanation), GCS M (Glasgow coma scale, best motor response), onset hour $\left( {\cos \left( {2\pi \frac{{hour_{onset} }}{24}} \right)} \right)$.

In the primary analysis of stroke prediction using logistic regression, random forest, support vector machines (SVM), and eXtreme Gradient Boosting (XGBoost), XGBoost had the highest predictive values (AUROC 0.994 [confidence interval; CI 0.991–0.997]) in the training cohort. In the test cohort, the XGBoost model also had the highest predictive value of the five machine learning approaches (AUROC 0.980 [CI 0.962–0.994]) (Table 2 and Fig. 1a, b). The SHapley Additive exPlanation (SHAP) summary plot revealed that the major predictive contributors for stroke were “sudden headache,” “upper limb paralysis,” “convulsion,” “sudden impaired consciousness or headache,” “systolic blood pressure,” “arrhythmia,” “conjugate deviation,” and “diastolic blood pressure” (Fig. 1c).

**Table 2:** Prehospital stroke prediction using machine learning.

Models	AUROC	Accuracy	Sensitivity	Specificity	F1-score
**Training cohort**					
XGBoost	0.994	0.978	0.990	0.947	0.985
Random forest	0.979	0.943	0.956	0.910	0.960
SVM (Radial basis function)	0.968	0.928	0.950	0.873	0.950
SVM (Linear)	0.889	0.835	0.915	0.627	0.889
Logistic regression	0.882	0.843	0.847	0.835	0.886
**Test cohort**					
XGBoost	0.980	0.952	0.986	0.864	0.967
Random forest	0.953	0.907	0.933	0.840	0.935
SVM (Radial Basis function)	0.935	0.900	0.933	0.815	0.931
SVM (Linear)	0.904	0.862	0.928	0.691	0.907
Logistic regression	0.886	0.828	0.828	0.827	0.874

*AUROC* area under the receiver operating characteristic curve, *XGBoost* eXtreme gradient boosting, *SVM* support vector machine.

### Prediction of stroke subcategories

We next analyzed the prediction algorithms for stroke subcategories (acute ischemic stroke [AIS] with /without LVO, intracranial hemorrhage [ICH], and subarachnoid hemorrhage [SAH]) using XGBoost, which was the best approach in the primary analysis. The machine learning-based prediction algorithms had high predictive values (test data, AUROC [CI], AIS with LVO 0.898 [0.848–0.939], AIS without LVO 0.882 [0.836–0.923], ICH 0.866 [0.817–0.911], SAH 0.926 [0.874–0.971]) (Table 3 and Supplementary Fig. S2). The SHAP summary plot of AIS with LVO revealed that the major contributors were “GCS V,” “onset hours,” “age,” “arrhythmia,” “hemiparalysis,” “systolic blood pressure,” “Japan Coma Scale (JCS),” and “minimum thermo-hydrological index (THI)” (Supplementary Fig. S2).

**Table 3:** Prehospital stroke subcategory prediction using XGBoost.

	AUROC	Accuracy	Sensitivity	Specificity	F1-score
**Training cohort**					
AIS with LVO	0.896	0.893	0.384	0.977	0.504
AIS without LVO	0.916	0.837	0.840	0.835	0.736
ICH	0.910	0.853	0.679	0.906	0.684
SAH	0.974	0.971	0.574	0.993	0.673
**Test cohort**					
AIS with LVO	0.898	0.897	0.488	0.964	0.571
AIS without LVO	0.882	0.814	0.810	0.815	0.703
ICH	0.866	0.834	0.618	0.901	0.636
SAH	0.926	0.952	0.333	0.985	0.417

*AUROC* area under the receiver operating characteristic curve, *XGBoost* eXtreme gradient boosting, *SVM* support vector machine, *AIS* acute ischemic stroke, *LVO* large vessel occlusion, *ICH* intracranial hemorrhage, *SAH* subarachnoid hemorrhage.

## Discussion

In this study of prehospital stroke prediction using machine learning, the algorithm using XGBoost had a high predictive value for strokes and stroke subcategories including LVO. It can be technically feasible that EMS personnel input required data for the predicting algorithm at the scene using tablet PCs and that the EMS and hospital personnel utilize the results of prediction, which may contribute to improving a quality of prehospital care.

Substantial investigations on prehospital predicting scales for strokes have been conducted with insufficient precision^5–7^. In a meta-analysis of the CPSS for stroke prediction (study n = 3, patient n = 1366), the CPSS had a summary AUROC of 0.813^5^. More recent investigation of the JUST score (patient n = 2236) documented AUROCs of 0.88 and 0.80 for stroke in the training (derivation) and test (validation) cohorts, respectively^6^. In accordance with these stroke predictions, insufficient predictive values of LVO prediction (AUROC 0.72–0.83) were documented in eight prehospital scales including the Rapid Arterial Occlusion Evaluation (RACE), Los Angeles Motor Scale, Cincinnati Stroke Triage Assessment Tool, Gaze-Face-Arm-Speech-Time, Prehospital Acute Stroke Severity, CPSS, Conveniently-Grasped Field Assessment Stroke Triage, and Face-Arm-Speech-Time plus severe arm or leg motor deficit test^7^. Thus, there is an unmet need for the improvement of the prediction precision for strokes/LVOs. The machine learning approach is a potential solution to improve precision, as we have demonstrated in this study. We found that the prediction algorithms using XGBoost had a high predictive value for strokes (AUROC 0.980) and stroke subcategories (AUROC 0.866–0.926) including LVOs (AUROC 0.898).

Substantial machine learning studies for diagnosis and prognosis in acute diseases have been documented^8–10^, while machine learning studies for strokes remain insufficient. To the best of our knowledge, investigations on predicting strokes by machine learning using a prehospital dataset have rarely been conducted. The majority of machine learning studies are focused on detecting strokes from computed tomography (CT) images, and machine learning studies focused on prehospital or hospital bedside prediction of strokes are limited^11^. In this study, we successfully developed prehospital stroke prediction algorithms using a machine learning approach with high precision. We found only one machine learning study that predicted LVOs with prehospital data. The study used 24 variables including prehospital data on 777 adult AIS patients (LVO n = 300) who underwent CT angiography or MR angiography and received reperfusion therapy within 8 h from symptom onset in a single center. They compared artificial neural network (ANN) and conventional stroke prediction scales including the Cincinnati Prehospital Stroke Severity Scale, Field Assessment Stroke for Emergency Destination, and RACE. They found that the ANN had a higher predictive value than the conventional scales (AUROC, ANN 0.823, conventional scales 0.740–0.796)^12^. In addition, prediction algorithms for LVOs using machine learning were documented by You et al., whose study was not a prehospital study but a hospital study^13^. Their study included 300 adult stroke patients (LVO n = 130) with 24 variables and compared the predictive values of XGBoost, logistic regression, random forest, and SVM. They found that XGBoost had the highest predictive value for LVOs (AUROC 0.809)^13^. In agreement with these findings, we also found that XGBoost had a higher predictive value for strokes compared to logistic regression, random forest, and SVM. Thus, XGBoost appears to be a better approach for this prediction.

Not only patients’ baseline factors, but also environmental factors, increased the risk of stroke^14^. In this study, of the 21 environmental factors screened, the onset timing factors including onset hour, day of the week (Monday), and minimum THI were analyzed. Stroke patients had earlier onset timing hours compared to non-stroke patients in the univariate analysis (Table 1). In addition, the SHAP analysis in the XGBoost for the identified onset hour of a stroke had an impact on the algorithms. Regarding the minimum THI, a high impact was identified in the SHAP analysis for LVOs. Few prehospital stroke scales include environmental factors; however, adding environmental factors may contribute to improving precision of prehospital stroke/stroke subcategory prediction using machine learning.

There were some limitations to this study. First, the study was a multicenter study, but conducted in a single urban region in Japan. Therefore, it remains unclear whether the algorism would have high predictive value in different areas with different backgrounds, and generalization. Second, we excluded pediatric patients, which may be a limiting factor of the present study; however, the frequency of stroke in pediatric patients was not common compared to adult and approximately 1.0 in 100,000 per year^15^. In addition, the predictive factors for pediatric stroke appears to be different compared to adults^15^. Further studies targeting pediatric stroke studies may develop different predicting algorithms. Third, we aimed for a better performance rather than limiting the number of predictors. Further development of algorithms with limited number of predictors to keep high predictive value would strengthen the study results and may lead to future implementation. Further studies including wider regions or a limited number of predictors may strengthen the findings for the prediction of strokes by machine learning.

In the conclusions**,** the prehospital stroke diagnostic algorithm using machine learning had a high predictive value for strokes and their subcategories including LVOs. This machine learning approach could potentially lead to precise stroke recognition in prehospital settings.

## Methods

### Study setting and patients

The current multicenter observational study was prospectively conducted in an urban area (Chiba city, population 1 million) in Japan, between September 2018 and September 2020. The Chiba City Fire Department covers this entire area with 26 ambulance squads, and has about 53,000 emergency dispatches per year. We included consecutive adult patients (≥ 20 years of age) with suspected stroke by EMS personnel who were subsequently transported to hospitals. There were 12 hospitals which can receive stroke patients in this entire area; all the 12 hospitals participated the study. We excluded pediatric patients, hypoglycemia proved by measurement of blood glucose, traumatic brain injury, drug abuse, undiagnosed patients who have not received diagnostic investigation based such as CT or magnetic resonance imaging (MRI). Of the 1778 patients screened, 332 who had missing diagnostic data or multiple entries were excluded and 1446 were analyzed (Supplementary Fig. S1).

The Chiba University Hospital Certified Clinical Research Review Board approved this study (No.2733) and waived the need for written informed consent, in conformity with the Ethical Guidelines for Medical and Health Research Involving Human Subjects in Japan. We posted information about this study in each ambulance. We promptly excluded the collected data when a patient or family indicated that they did not wish to participate in this study.

### Data collection and definition

Data on variables for stroke prediction in the prehospital setting, including symptoms, physiological data, and medical history, were collected (Supplementary Table S1). The variables cover each of the scoring components of the National Institutes of Health Stroke Scale^16^. Since onset timing (daily, especially Monday, and hourly) and meteorological conditions potentially altered the risk of stroke^14,17^, onset time and weather data were added to the analysis.

Stroke is defined based on the National Institute of Neurdological Disorders and Stroke III^18^. Stroke is further subclassified into AIS with LVO, AIS without LVO, ICH, and SAH. LVO is defined as acute occlusion of the internal carotid artery, M1 or M2 portion of the middle cerebral artery, and the basilar artery. Board-certified neurologists in registered hospitals made the diagnoses based on examinations including CT, MRI, CT angiography, and MR angiography.

### Missing values

We used domain knowledge to impute missing values first. The mutually imputed pairs or groups of features based on the knowledge were as follows: (i) conjugate deviation and visual field defects, (ii) dysarthria and facial paralysis, (iii) aphasia, best verbal response of the GCS, the JCS, and consciousness-related features.

For the remaining missing values of highly correlated variables (correlation coefficient > 0.7), multivariate imputation was applied between the variables using a regressor model, in which each feature with missing values is modeled as a function of other features. The mutually imputed pairs or groups of features were as follows: (1) systolic and diastolic blood pressure, (2) left and right pupil sizes, (3) left and right pupillary light reflex, (4) the GCS, the JCS, and consciousness-related features, and (5) paralysis-related features. Other missing values of the numerical features were imputed with each median value. For any other categorical attributes, the missing values were replaced with a new subcategory “Unknown”.

Note that XGBoost can handle missing values, unlike the other methods. Therefore, we trained two XGBoost models, one with the data before and the other after applying imputation, and confirmed that the imputation did not cause a decrease in performance (paired sampled t-test’s *P*-value = 0.268 for tenfold cross validation scores). For the purpose of comparison between the other models (logistic regression, random forest, SVM) and XGBoost, we used the imputed data for all analyses in this study.

### Feature selection

Among the 59 features collected by EMS personnel and onset time variables, 51 features were selected after primitive cleaning of the feature candidates with a large fraction (over 40%) of missing values (2 features) or low variance (5% cutoff, 6 features).

For the meteorological features, we calculated Pearson’s chi-square values for all pairs between the stroke category and the 21 meteorological features (Supplementary Methods) and selected the features with *P*-values < 0.05 as feature candidates. These selected meteorological features were further reduced in number by a forward stepwise selection method, in which only features that improved the performance of the model were selected by repeatedly including the features one by one. Finally, only one meteorological feature, the ‘minimum THI on the onset day’ was selected.

Because we aimed to improve performance, rather than simply limit the number of predictors, we included 52 features for all analyses in this study.

### Statistical analysis

Of the 1446 patients analyzed, 1156 (80%) were randomly included in a training cohort as a derivation cohort, and 290 (20%) were in a test cohort as a validation cohort. First, we developed the binary classification models for stroke classification as a primary outcome based on five common machine learning algorithms: logistic regression, random forest, SVM with linear or radius basis function kernels, and XGBoost. Then, as a secondary outcome, we built a multi-class classification model to predict each stroke category of AIS with/without LVO, ICH, and SAH. Based on the primary analysis that found XGBoost to be superior, we chose XGBoost for the secondary outcome analysis.

The parameters of the five machine learning models were selected by using the grid search method, in which we further split the training cohort into five folds, trained each of the five sets of data, and selected the parameters of the model that performed the best. It should be noted that the data are imbalanced. More weight must be set toward the minor classes in any model when the loss functions are calculated so that the major and minor classes are fairly evaluated.

The performance of the models was measured in terms of the AUROC as a superior metric, as well as sensitivity, specificity, and the F1 score. We used the SHAP algorithm^19^ of the XGBoost model to interpret the contributions of each feature to the predictive model. In the algorithm, the SHAP value was computed by a difference in model output resulting from the inclusion of a feature in the algorithm, providing information on each feature’s impact on the output. In the SHAP summary plots, every violin plot is composed of all data points of each feature with a higher value being redder, and a lower value being bluer. The violin plots are aligned with the SHAP value along the x-axis. Thus, a redder/bluer violin plot on the right side (i.e., higher positive SHAP value) suggests that the higher/lower the values of the feature are, the more the model predicts towards positive/negative impact.

Data are expressed as medians (interquartile ranges) for continuous values and absolute numbers and percentages for categorical values. Two-tailed *P*-values < 0.05 were considered significant. Analyses were performed using Python 3.7.6 packages (Scikit-learn 0.23.2, XGBoost 1.1.1, Pandas 1.1.5, and NumPy 1.19.2) to construct the machine learning models. The Python packages including Scikit-learn, XGBoost, Pandas, Numpy, and Matplotlib, and the SHAP package are all open-source packages. Permission to use these packages is granted, free of charge, to any person (Python License: https://docs.python.org/3/license.html, SHAP: https://github.com/slundberg/shap/blob/master/LICENSE). All figures in this study were drawn using the visualization package in Python, Matplotlib (3.3.4)^20,21^.

## Supplementary Information

Supplementary Information.

## Data Availability

The datasets used and analyzed during our study are available from the corresponding author upon reasonable request.
