# Supplementary material for: A prehospital diagnostic algorithm for strokes using machine learning: a prospective observational study
Source: Sci Rep. 2021 Oct 15;11:20519. doi: 10.1038/s41598-021-99828-2 (PMC8521587; doi:10.1038/s41598-021-99828-2)
Supplement: Supplementary file 1 — Supplementary Information. [file 41598_2021_99828_MOESM1_ESM.pdf]

Supplemental materials

Supplementary Information

Expanded Methods

Supplementary Table S1. Collected variables

Supplementary Table S2. Baseline characteristics and clinical outcomes of the test cohort

Supplementary Figure S1. Study flowchart

Supplementary Figure S2. Receiver operating characteristic curve of stroke subcategories and the SHAP value of prehospital large vessel occlusion prediction
Supplementary Methods

Meteorological data

Daily weather conditions were added as explained features of the model to incorporate the previously reported relationship between stroke and weather[14]. Hourly weather data from the Chiba observational station were obtained from the Japan Meteorological Agency (https://www.jma.go.jp/jma/indexe.html). We used hourly ambient temperature, air pressure, and relative humidity data to generate new features such as the thermo-hydrological index (THI). The THI (with units of °C) is given by

$$THI = T_a - 0.55(1 - 0.01RH)(T_a - 14.5),$$

where T_a is ambient temperature and RH is relative humidity.[22]

Note that we filled 298 missing onset data times with the discovery data times (or with the command data times if the discovery datetime was also not available), in order to merge the prehospital data with the weather data without missing any weather data.

We used 21 meteorological features as primitive candidates for further selection (see Section ***Feature selection***) and listed them below.

THI_min	Minimum THI
THI_max	Maximum THI
THI_mean	Mean THI
THI_ptp	THI range
pressure_min	Minimum air pressure
pressure_max	Maximum air pressure
pressure_mean	Mean air pressure
pressure_ptp	Air pressure range
humidity_min	Minimum relative humidity
humidity_max	Maximum relative humidity
humidity_mean	Mean relative humidity
THI_diff_days	Mean THI difference from the previous day
pressure_diff_days	Mean air pressure difference from the previous day
humidity_ptp	Relative humidity range
humidity_diff_days	Mean relative humidity difference from the previous day
THI_5h-1h	THI difference between at five o'clock and at one o'clock on the date
THI_diff_previous_2days	THI difference from the average two days prior to the onset date
THI_diff_previous_3days	THI difference from the average three days prior to the onset date
THI_diff_previous_4days	THI difference from the average four days prior to the onset date
THI_diff_previous_5days	THI difference from the average five days prior to the onset date
THI_diff_previous_6days	THI difference from the average on the six days prior to the onset date

Onset time data

We split the onset time variable into two new features, that is, transformed it to a sine and cosine function to express its periodic nature. Each variable is defined as:

$$hour_{cos} = \cos\left(2\pi \frac{hour_{onset}}{24}\right),$$

$$hour_{sin} = \sin\left(2\pi \frac{hour_{onset}}{24}\right).$$

For example, $hour_{cos} > 0$ means 0H-12H and $hour_{sin} < 0$ means 6H-18H.

References

22. Sueda Y. *et al*. Effects of meteorological conditions on the risk of ischemic stroke events in patients treated with alteplase--hews-tpa. *J Stroke Cerebrovasc Dis.* 24, 1500-1505 (2015).

Supplementary Table S1. Collected variables

Age	Symptoms
Sex	Headache
Body mass index	Numbness
Activity of daily life	Nausea
	Vomiting
Past medical history	Dizziness
Atrial fibrillation	Convulsion
Hypertension	Cardiac arrest
Diabetes mellitus	Arrhythmia
Intracranial hemorrhage	Consciousness disturbance
Cerebral infraction	Miosis
Epilepsy	Mydriasis
Psychiatric disorder	Upper limb paralysis
Warfarin, DOAC, and antiplatelet	Lower limb paralysis
	Aphasia
Vital signs	Dysarthria
Japan coma scale	Conjugate deviation
Glasgow coma scale	Visual field defects
Heart rate	Facial palsy
Systolic blood pressure	Ataxia
Diastolic blood pressure	Sensory impairment
Body temperature	Unilateral spatial neglect
SpO_2	
	Weather data
Onset timing (hour)	Thermo-hydrological index
Onset timing (Monday)	Mean air pressure
	Air pressure difference

DOAC (direct oral anticoagulant)

Supplementary Table S2. Baseline characteristics and clinical outcomes of the test cohort

	Stroke (n=209)	Non-stroke (n=81)	*P* value
Age, years	73.0 (61.0-81.0)	74.0 (61.0-82.0)	.815
Male sex, n (%)	124 (59.3)	46 (56.8)	.794
Past medical history			
Atrial fibrillation, n (%)	16 (7.7)	9 (11.1)	.638
Hypertension, n (%)	94 (45.0)	30 (37.0)	.211
Diabetes mellitus, n (%)	32 (15.3)	13 (16.0)	.621
Intracranial hemorrhage, n (%)	11 (5.3)	4 (4.9)	.642
Cerebral infarction, n (%)	48 (23.0)	19 (23.5)	.720
Epilepsy, n (%)	3 (1.4)	3 (3.7)	.389
Psychiatric disorder, n (%)	6 (2.9)	4 (4.9)	.686
Anticoagulant, n (%)	22 (10.5)	9 (11.1)	.985
Vital signs			
Heart rate	80.0 (70.0-96.0)	88.0 (70.0-98.0)	.167
Arrhythmia, n (%)	44 (21.1)	11 (13.6)	.009
Systolic blood pressure	173.0 (151.0-196.0)	170.0 (142.0-187.0)	.109
Diastolic blood pressure	95.0 (84.0-110.0)	94.0 (80.0-108.0)	.534
Body temperature	36.4 (36.2-36.8)	36.5 (36.2-37.0)	.074
Japan Coma Scale=0, n (%)	86 (41.1)	46 (56.8)	.445
Glasgow Coma Scale			
Eye opening=4, n (%)	143 (68.4)	68 (84.0)	.063
Best verbal response=5, n (%)	95 (45.5)	49 (60.5)	.201
Best motor response=6, n (%)	151 (72.2)	61 (75.3)	.216
Symptoms			
Vomiting, n (%)	31 (14.8)	9 (11.1)	.645
Dizziness, n (%)	15 (7.2)	12 (14.8)	.108
Convulsion, n (%)	2 (1.0)	10 (12.3)	<.001
Upper limbs paralysis, n (%)	85 (40.7)	30 (37.0)	.665
Lower limbs paralysis, n (%)	100 (47.8)	28 (34.6)	.056
Hemiparalysis, n (%)	51 (24.4)	5 (6.2)	.001
Conjugate deviation, n (%)	15 (7.2)	7 (8.6)	.889
Visual field defects, n (%)	1 (0.5)	2 (2.5)	.283
Facial palsy, n (%)	37 (17.7)	8 (9.9)	.204
Ataxia, n (%)	23 (11.0)	8 (9.9)	.824
Sensory impairment, n (%)	29 (13.9)	4 (4.9)	.066
Aphasia, n (%)	23 (11.0)	8 (9.9)	.927
Dysarthria, n (%)	69 (33.0)	11 (13.6)	.001
Unilateral spatial neglect, n (%)	2 (1.0)	0 (0.0)	.627
Onset timing Monday, n (%)	27 (12.9)	9 (11.1)	.826
Onset timing (hour)	12.0 (8.0-17.0)	13.0 (8.0-18.0)	.523
Minimum THI	13.7 (7.6-18.8)	11.7 (7.6-18.3)	.411

THI (thermo-hydrological index)
Data are presented as median and interquartile range for continuous variables.
P-values were calculated using Pearson’s chi-square test or the Mann–Whitney U test.

Supplementary Figure S1. Study flowchart

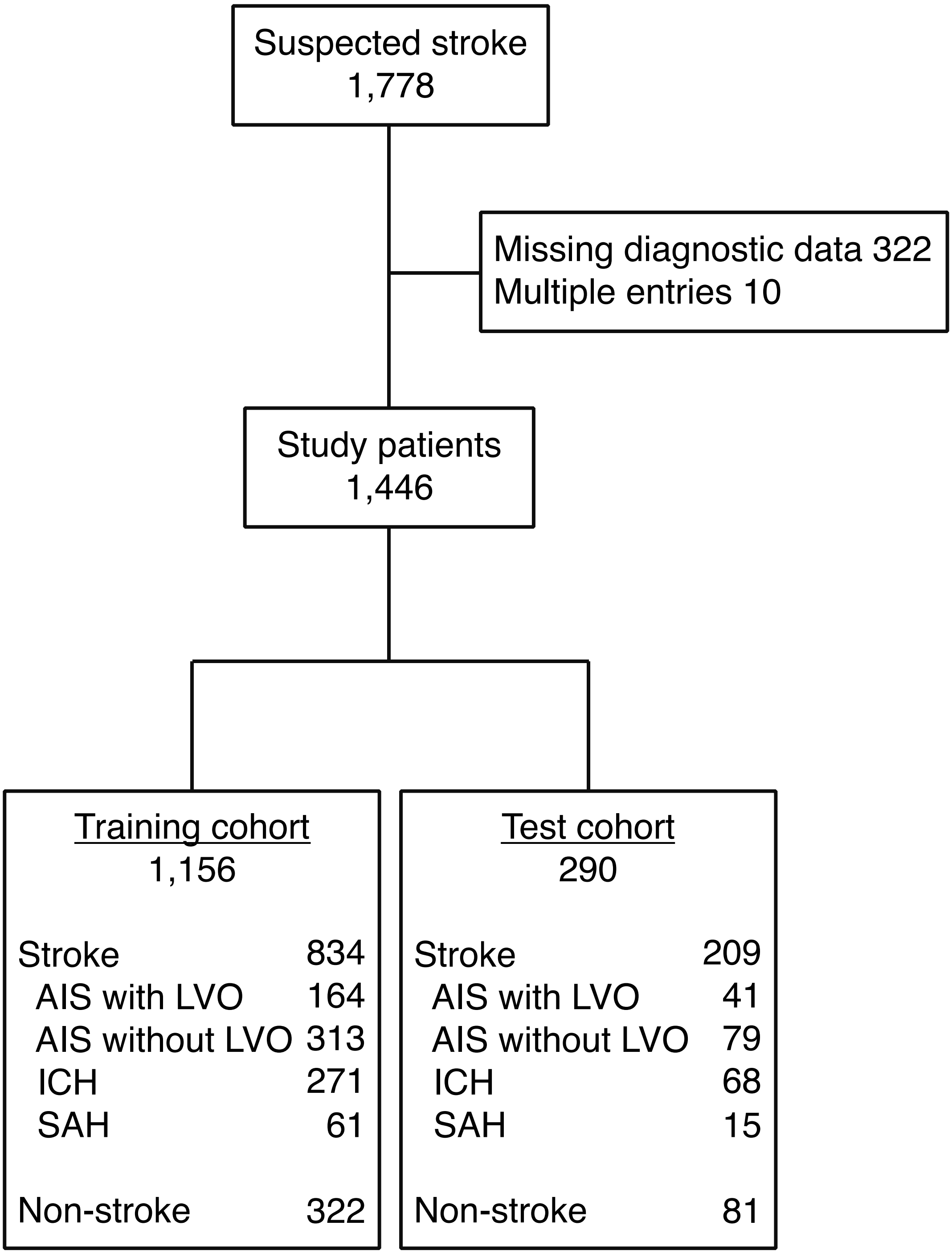

AIS (acute ischemic stroke), LVO (large vessel occlusion), ICH (intracranial hemorrhage), SAH (subarachnoid hemorrhage)

Supplementary Figure S2. Receiver operating characteristic curve of stroke subcategories and the SHAP value of prehospital large vessel occlusion prediction

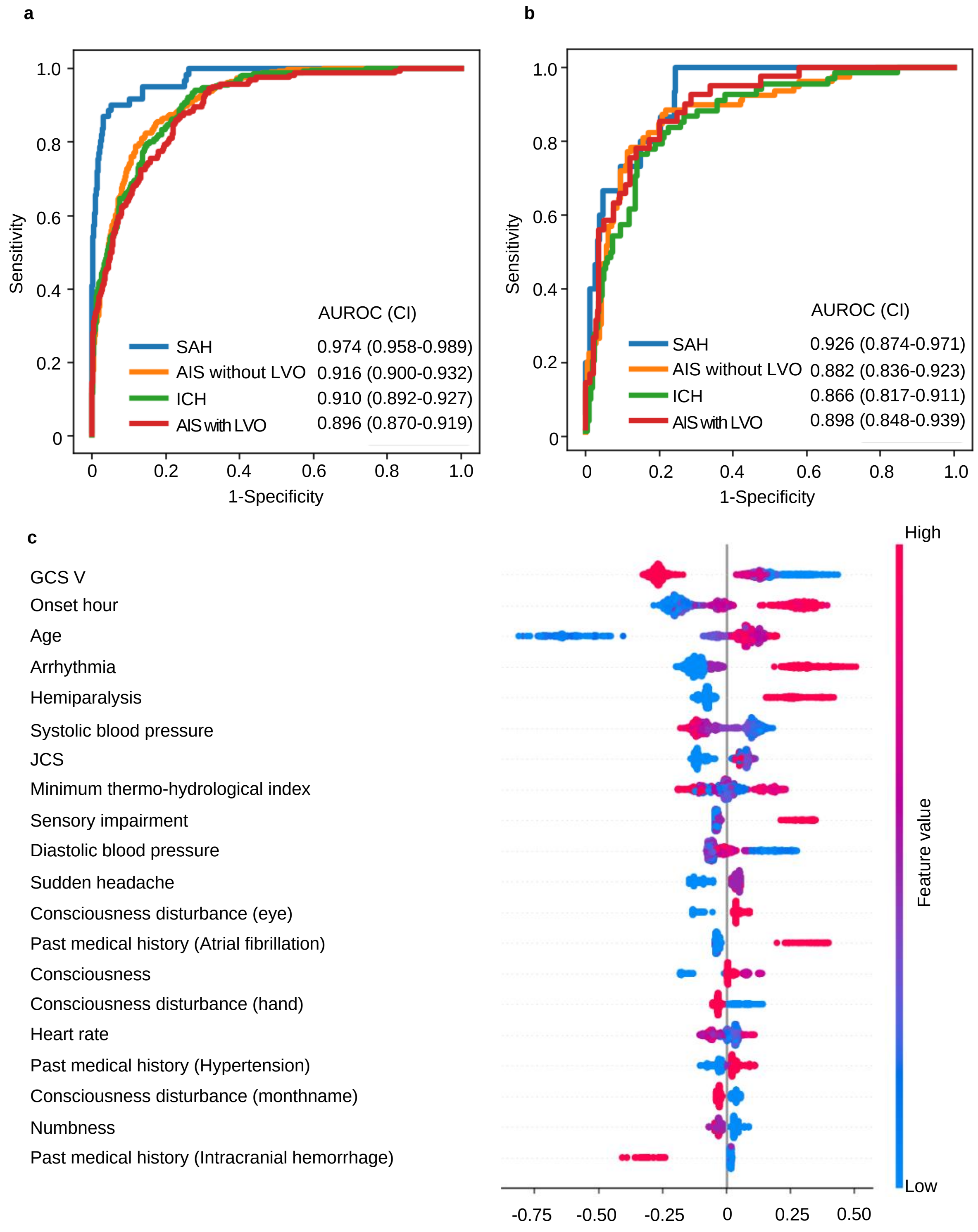

a. Training cohort. **b.** Test cohort. **c.** SHAP value of large vessel occlusion. SHAP (SHapley Additive exPlanation), Onset hour ($\cos\left(2\pi\frac{hour_{onset}}{24}\right)$,), GCS V (Glasgow coma scale, best verbal response), JCS (Japan coma scale)
